# Surveillance of antimicrobial-resistant *Escherichia coli* in Sheltered dogs in the Kanto Region of Japan

**DOI:** 10.1038/s41598-021-04435-w

**Published:** 2022-01-14

**Authors:** Akihisa Hata, Noboru Fujitani, Fumiko Ono, Yasuhiro Yoshikawa

**Affiliations:** 1 Faculty of Veterinary Medicine, Okayama University of Science, Ikoino-oka 1-3, Imabari, Ehime 7948555 Japan; 2 Biomedical Science Examination and Research Center, Okayama University of Science, Ikoino-oka 1-3, Imabari, Ehime 7948555 Japan

**Keywords:** Policy and public health in microbiology, Zoology

## Abstract

There is a lack of an established antimicrobial resistance (AMR) surveillance system in animal welfare centers. Therefore, the AMR prevalence in shelter dogs is rarely known. Herein, we conducted a survey in animal shelters in Chiba and Kanagawa prefectures, in the Kanto Region, Japan, to ascertain the AMR status of *Escherichia coli*  (*E. coli*) prevalent in shelter dogs. *E. coli* was detected in the fecal samples of all 61 and 77 shelter dogs tested in Chiba and Kanagawa, respectively. The AMR was tested against 20 antibiotics. *E. coli* isolates derived from 16.4% and 26.0% of samples from Chiba and Kanagawa exhibited resistance to at least one antibiotic, respectively. *E. coli* in samples from Chiba and Kanagawa prefectures were commonly resistant to ampicillin, piperacillin, streptomycin, kanamycin, tetracycline, and nalidixic acid; that from the Kanagawa Prefecture to cefazolin, cefotaxime, aztreonam, ciprofloxacin, and levofloxacin and that from Chiba Prefecture to chloramphenicol and imipenem. Multidrug-resistant bacteria were detected in 18 dogs from both regions; β-lactamase genes (*blaTEM*, *blaDHA-1*, *blaCTX-M-9* group *CTX-M-14*), quinolone-resistance protein genes (*qnrB* and *qnrS*), and mutations in quinolone-resistance-determining regions (*gyrA* and *parC*) were detected. These results could partially represent the AMR data in shelter dogs in the Kanto Region of Japan.

## Introduction

Companion animals may be reservoirs and spillover hosts for resistant bacteria^1–5^, raising concerns of health risks posed by resistant bacteria harbored by companion animals to humans^6–9^. Infection with drug-resistant bacteria not only prolongs treatment periods but is also life-threatening for the elderly and individuals with a weakened immune system. A global action plan concerning bacterial drug resistance was adopted at the World Health Organization general meeting in 2015^10^. Subsequently, in 2016, the Japanese Government presented the antimicrobial drug resistance (AMR) action plan^11^. In 2019, the United Nations Interagency Coordination Group on Antimicrobial Resistance released a report calling for urgent action to avoid an AMR crisis^12^. The report included the following aims: (a) monitoring AMR and administration of antimicrobial drugs, (b) identification of indicators of change in drug resistance, and (c) further expansion and development of the action plan. To accomplish these aims, AMR surveillance in several different fields is required, including human and veterinary medicine, agriculture, animal husbandry, and wild animal populations.

In Japan, sheltered dogs and cats should undergo microbiological testing for parasites, protozoans, and viruses before adoption^13^; however, an AMR surveillance system for dogs and cats in shelters has not been established. Therefore, the prevalence of AMR in shelter dogs is rarely known. In this study, we conducted a survey in two animal shelter centers in the Kanto Region to ascertain the status of AMR in *Escherichia coli* carriage in shelter dogs.

As medicines for companion animals in Japan include antibiotic agents specific for both animals and humans, various agents must be tested. In Japan, public and large-scale AMR surveys in livestock and human medicine are ongoing, including the Japanese Veterinary Antimicrobial Resistance Monitoring System (JVARM) managed by the Ministry of Agriculture, Forestry and Fisheries of Japan (MAFF), and the Japan Nosocomial Infections Surveillance (JANIS) managed by the Japan Ministry of Health, Labor, and Welfare. We considered that it is desirable to employ the same antibacterial agents that are being used by the JVARM and JANIS for AMR monitoring in this study. These results will make up for the lack of AMR data in shelter dogs in Japan.

## Results

### Antimicrobial drug susceptibility

*E. coli* was detected in the feces of all 138 dogs tested (61 from Chiba, 77 from Kanagawa). The following 20 antibiotics were selected for monitoring drug resistance in *E. coli*: ampicillin (ABPC), piperacillin (PIPC), tazobactam/piperacillin (TAZ/PIPC), cefazolin (CEZ), cefmetazole (CMZ), cefotaxime (CTX), ceftazidime (CAZ), cefepime (CFPM), aztreonam (AZT), imipenem (IPM), meropenem (MEPM), streptomycin (SM), kanamycin (KM), gentamicin (GM), amikacin (AMK), tetracycline (TC), ciprofloxacin (CPFX), levofloxacin (LVFX), nalidixic acid (NA), and chloramphenicol (CP). The breakpoint of resistance was based on Clinical and Laboratory Standards Institute (CLSI) M100-S24 criteria^14^.

Drug-susceptibility testing in the 61 *E. coli* isolates from Chiba revealed that the isolates derived from 10 dogs (16.4%) were resistant to at least one antibacterial drug among ABPC, PIPC, IPM, SM, KM, TC, NA, and CP (Table 1). None of the isolates exhibited resistance to TAZ/PIPC, CEZ, CMZ, CTX, CAZ, CFPM, AZT, MEPM, GM, AMK, CPFX, or LVFX. Some isolates exhibited intermediate resistance to CEZ, AZT, MEPM, AMK, and LVFX.

**Table 1:** Drug susceptibility of *Escherichia coli* isolated from shelter dogs in the Kanto Region, Japan.

Antimicrobial agent	Monitoring in JVARM and/or JANIS		CLSI breakpoint (mm)^a^		AMR (%)									
					This study								Previous study	
					Chiba (n = 61)				Kanagawa (n = 77)				healthy dogs^b^	ill dogs^b^
	JVARM	JANIS	I	R	I	95% CI	R	95% CI	I	95% CI	R	95% CI	R (%)	R (%)
Ampicillin	○	○	14–16	≤ 13	1.6	0–9.6	11.5	5.4–22.1	6.5	2.5–14.7	11.7	6.1–21.0	33.8	55.3
Piperacillin		○	18–20	≤ 17	1.6	0–9.6	9.8	4.2–20.2	2.6	0.2–9.5	7.8	3.3–16.3	N	N
Tazobactam/Piperacillin		○	18–20	≤ 17	0.0		0.0		1.3	0–7.7	0.0		N	N
Cefazolin	○	○	20–22	≤ 19	21.3	12.8–33.3	0.0		29.9	20.8–40.9	7.8	3.3–16.3	19.2	31.2
Cefmetazole		○	13–15	≤ 12	0.0		0.0		1.3	0–7.7	0.0		N	N
Cefotaxime	○	○	23–25	≤ 22	0.0		0.0		5.2	1.6–13.0	1.3	0–7.7	13.2	26.1
Ceftazidime		○	18–20	≤ 17	0.0		0.0		3.9	0.9–11.3	0.0		N	N
Cefepime		○	−	≤ 18	-		0.0		-		0.0		N	N
Aztreonam		○	18–20	≤ 17	1.6	0–9.6	0.0		0.0		2.6	0.2–9.5	N	N
Imipenem		○	20–22	≤ 19	0.0		1.6	0–9.6	1.3	0–7.7	0.0		N	N
Meropenem		○	20–22	≤ 19	1.6	0–9.6	0.0		0.0		0.0		0.0	0.0
Streptomycin	○		12–14	≤ 11	29.5	19.5–42.0	4.9	1.1–14.0	66.2	55.1–75.8	13.0	7.0–22.5	19.2	29.6
Kanamycin	○		14–17	≤ 13	16.4	9.0–27.8	4.9	1.1–14.0	16.9	10.0–26.9	2.6	0.2–9.5	5.3	6.5
Gentamicin	○		13–14	≤ 12	0.0		0.0		1.3	0–7.7	0.0		3.3	14.1
Amikacin		○	15–16	≤ 14	4.9	1.1–14.0	0.0		2.6	0.2–9.5	0.0		N	N
Tetracycline	○		12–14	≤ 11	1.6	0–9.6	9.8	4.2–20.2	0.0		2.6	0.2–9.5	16.6	28.1
Ciprofloxacin	○		16–20	≤ 15	0.0		0.0		5.2	1.6–13.0	2.6	0.2–9.5	18.5	43.2
Levofloxacin		○	0.25–1	≥ 2	3.3	0.2–11.8	0.0		7.8	3.3–16.3	2.6	0.2–9.5	N	N
Nalidixic acid	○		14–18	≤ 13	3.3	0.2–11.8	1.6	0–9.6	0.0		5.2	1.6–13.0	27.8	61.8
Chloramphenicol	○		13–17	≤ 12	1.6	0–9.6	6.6	2.1–16.1	1.3	0–7.7	0.0		4.6	12.6

*95% CI* 95% confidence interval, *I* intermediate, *R* resistant, *N* not subject to survey, *JVARM* Japanese Veterinary Antimicrobial Resistance Monitoring System, *JANIS* Japan Nosocomial Infections Surveillance, *CLSI* Clinical and Laboratory Standards Institute, *AMR* antimicrobial resistance.

^a^Disk diffusion zone diameter interpretive criteria (mm). Only LVFX was tested using the broth microdilution method; minimum inhibitory concentration (MIC) interpretive criteria (μg/mL).

^b^FY 2018 antimicrobial resistance monitoring survey of bacteria derived from healthy companion animals (dogs and cats), Ministry of Agriculture, Forestry and Fisheries of Japan (MAFF).

Drug-susceptibility testing in the 77 *E. coli* isolates from Kanagawa revealed that the isolates derived from 20 dogs (26.0%) were resistant to at least one antibacterial drug among ABPC, PIPC, CEZ, CTX, AZT, SM, KM, TC, CPFX, and NA (Table 1). None of the isolates exhibited resistance to TAZ/PIPC, CMZ, CAZ, CFPM, IPM, MEPM, GM, AMK, LVFX, or CP. Some isolates exhibited intermediate resistance to TAZ/PIPC, CMZ, CAZ, IPM, GM, AMK, LVFX, and CP.

ABPC-, PIPC-, SM-, KM-, TC-, and NA-resistant *E. coli* were commonly found in dogs from Chiba and Kanagawa prefectures. CEZ-, CTX-, AZT-, and fluoroquinolone (CPFX and LVFX)-resistant *E. coli* were found only in Kanagawa Prefecture. CP- and IPM-resistant *E. coli* were found only in Chiba Prefecture.

The chi-square test of sex-related differences in the ratio of susceptible (S), intermediate (I), and resistant (R) results of the antimicrobial susceptibility test revealed no significant differences between males and females for any of the antibacterial agents.

Multidrug-resistant *E. coli* was detected in 18 dogs, with resistance to as many as six drugs in 1 dog and five drugs in 5 dogs. The patterns of multidrug resistance are shown in Table 2a.

**Table 2:** Pattern of multi-drug resistance and detected antimicrobial resistance genes in *E. coli.*

	Dog sample number																	
	Chiba								Kanagawa									
	16C1	16C26	16C37	16C42	16C43	16C44	17C2	17C16	16K18	16K21	17K2	17K8	17K12	17K20	17K27	17K36	17K49	17K55
**(a) Pattern of multi-drug resistance in *E. coli***																		
Antibiotic																		
Ampicillin	R	R	R	R	R	R		R	R	R	R	R	R	R	R		R	R
Piperacillin	I	R	R	R		R		R	R	I		I	R	R	R		R	R
Tazobactam/Piperacillin												I						
Cefazolin		I		I			I	I	R	R	R	R	R	I	I	I	I	R
Cefmetazole										I								
Cefotaxime									R	I	I	I						
Ceftazidime										I		I				I		
Cefepime																		
Aztreonam							I					R				R		
Imipenem							R											
Meropenem							I											
Streptomycin	I		I		R	R		R	I	I	I	I	R	R		R	I	
Kanamycin	R	R		R										R		I		
Gentamicin														I				
Amikacin																		
Tetracycline	R	R		R	R	R							R	R				
Ciprofloxacin															R	I	R	
Levofloxacin		I		I					R						I		R	
Nalidixic acid							R		R								R	
Chloramphenicol	R	R		R	R													
**(b) Detected antimicrobial resistance genes in *E. coli***																		
Resistance mechanism																		
β-lactamase	N.T	*bla TEM*	*bla TEM*	*bla TEM*	N.D	*bla TEM*	N.D	N.D	*bla CTX-M-9 group* *CTX-M-14*	*bla DHA-1*	N.D	N.D	*bla TEM*	*bla TEM*	*bla TEM*	N.D	*bla TEM*	*bla TEM*
Aminoglycoside resistance 16S rRNA methylases	N.T	N.D	N.D	N.D	N.D	N.D		N.D	N.D	N.D	N.D	N.D	N.D	N.D		N.D	N.D	
Aminoglycoside modifying enzyme	N.T	N.D	N.D	N.D	N.D	N.D		N.D	N.D	N.D	N.D	N.D	N.D	N.D		N.D	N.D	
Mutation of the quinolone resistance-determining regions		N.D		N.D			N.D		83S → L, 87D → Y in *gyrA* 80S → I in *parC*						N.D	N.D	83S → L, 87D → N in *gyrA* 80S → I in *parC*	
Quinolone resistance protein		*qnrS*		*qnrS*			*qnrB*		N.D						N.D	N.D	N.D	

*I* intermediate, *R* resistant, *N.D.* not detected, *N.T.* not tested.

### Detection of antimicrobial resistance genes

Drug-resistance genes detected in *E. coli* isolates that showed multidrug resistance are shown in Table 2b. In 17 isolates (originally 18 samples, but one sample could not be tested due to poor growth) that showed resistance or intermediate resistance to any of the β-lactams reagents, *blaTEM* (9 samples) and *blaDHA-1* (1 sample) were detected. In five third-generation cephalosporin (CTX and CAZ)-resistant or intermediate-resistant isolates, the *blaCTX-M-9* group *CTX-M-14* (1 sample) was detected. No carbapenemase gene was detected in isolates resistant to IPM. No aminoglycoside resistant 16S rRNA methylases genes and aminoglycoside-modifying enzyme genes were detected in 14 aminoglycoside (SM, KM, and GM)-resistant or intermediate-resistant isolates (originally 15 samples, but one sample could not be tested due to poor growth). In seven quinolone-resistant or intermediate-resistant isolates, *qnrB* (1 sample) and *qnrS* (2 samples) were detected. Mutations in quinolone-resistance-determining regions (QRDR), 83 serine (S) and 87 aspartic acid (D) of the gyrA sequence and 80S of the parC sequence (2 samples), were detected. The first sample showed mutations of 83S to leucine (L) and 87D to tyrosine (Y) in gyrA and 80S to isoleucine (I) in parC. In the second sample, 83S was mutated to L and 87D was mutated to asparagine (N) in gyrA, and 80S to isoleucine (I) in parC. *blaTEM* was commonly detected in Chiba and Kanagawa prefectures. *qnrB* and *qnrS* were detected only in Chiba Prefecture, and the *blaCTX-M-9* group *CTX-M-14*, *blaDHA-1*, and quinolone-resistant mutations were detected only in Kanagawa Prefecture.

## Discussion

Drug-resistant *E. coli* was detected in some of the shelter dogs surveyed in this study. In addition, resistance genes related to the resistance mechanism were identified. First, we compared drug-susceptibility testing results with data available in Japan. Most of the canine AMR data currently reported in Japan are from animal patients who visited veterinary clinics for the treatment of some diseases. Other than those released by the MAFF in 2020^15^, almost no AMR survey data are available for non-patient companion animals. Table 1 compares the results of our study with the drug-resistance rates of dog rectal swab-isolated *E. coli* reported by the MAFF. The MAFF survey also included dogs taken to a veterinary hospital in 2017 (ill dogs) and 2018 (healthy dogs), which overlaps with our survey period (2016–2017). Regarding common antibacterial agents tested in our study and the MAFF survey (ABPC, CEZ, CTX, MEPM, SM, KM, GM, TC, CPFX, NA, and CP), the antibiotic resistance rate observed in sheltered dogs was mostly lower than that in healthy dogs in the MAFF survey. In the samples obtained from Chiba, the 95% confidence interval (95% CI) range of the antibiotic resistance rates against ABPC, CEZ, CTX, MEPM, SM, GM, CPFX, and NA in sheltered dogs was lower than that in healthy dogs in the MAFF survey (Table 1). The 95% CI range of the resistance rates against KM, TC, and CP in sheltered dogs overlapped with that in healthy dogs in the MAFF survey. In the samples obtained from Kanagawa, the 95% CI range of the antibiotic resistance rates against ABPC, CEZ, CTX, MEPM, GM, TC, CPFX, NA, and CP in sheltered dogs was lower than that in healthy dogs in the MAFF survey (Table 1). The 95% CI range of the resistance rates against KM and SM in sheltered dogs overlapped with that in healthy dogs in the MAFF survey. In the MAFF survey, the resistance rates in healthy dogs were lower than those in sick dogs^15^. The 95% CI range of the resistance rates against KM and CP in the samples from Chiba and against KM in the samples from Kanagawa overlapped with that in the sick dogs in the MAFF survey (Table 1). The use of β-lactam antibiotics and fluoroquinolone antibiotics in veterinary medicine has been reported to promote an increase in the number of drug-resistant *E. coli* isolates^1,16^. Sheltered dogs include abandoned and stray dogs; presumably, these dogs are less exposed to veterinary medical facilities and the administration of antibacterial drugs than dogs in households. This may explain the lower drug-resistance rate observed in our study than in the MAFF survey.

Next, the results of the identification of drug-resistance genes were compared with data from Japan and other countries. Several types of β-lactamase genes, QRDR mutations, and quinolone-resistant protein genes were detected in *E. coli* from shelter dogs. β-Lactamase genes, *blaTEM*, *blaCTX-MTX-M-14*, and *blaDHA*, were detected. These are genes that are reportedly detected in the intestinal bacteria of humans, farm animals, and companion animals^17–21^. A 2016 study of sheltered dogs and cats in Osaka, Japan, reported that many of these resistance genes are detected in cephalosporin-resistant *E. coli*^22^. As quinolone-resistance mechanisms, QRDR mutations and quinolone-resistance proteins (*qnrB* and *qnrS*) were detected. Furthermore, β-lactamase genes, which are also involved in resistance mechanisms, have been detected in humans, farm animals, and companion animals^17,23–25^. The quinolone-resistant mechanisms have been predominantly detected in a survey of *E. coli* in shelter dogs and cats in Osaka from 2016 to 2017^26^. Therefore, the drug-resistance mechanism in *E. coli* detected in this study was of the type that has been reportedly detected in the intestinal bacteria of dogs in Japan and abroad.

In conclusion, the rates of resistance to various antibiotics among the *E. coli* isolated from shelter dogs in the animal welfare centers in Chiba and Kanagawa prefectures were mostly lower than those in the healthy and sick domestic dogs in Japan, surveyed at almost the same time^15^. The detected resistance genes presented the same trend as those reported in shelter dogs in the same years in Japan^22,26^. As several studies have already mentioned, drug-resistant bacteria in companion animals can be a health risk to humans^6–9^. AMR surveillance in companion animals, including shelter dogs, for which there is a lack of data, needs to be widely conducted to accurately assess the AMR prevalence in Japan. The present results will make up for the lack of AMR data in shelter dogs.

## Methods

### Sampling of dog feces

This study was conducted in accordance with the principles of the ARRIVE guidelines. Feces from sheltered dogs were used, and no invasive treatment was performed on the dogs; therefore, the study did not require ethics approval.

The required sample size (n) was calculated at a 95% confidence level using the formula and parameters below. The proportion of AMR (P) in the population was estimated as 10%, based on the results of the preliminary survey. The margin of error (δ) was 0.08. The required sample size was estimated to be 54.

$$n=\frac{{1.96}^{2}\times P\times (1-P)}{{\updelta }^{2}}$$

Between 2016 and 2017, we collected feces from 61 and 77 dogs housed in two public animal welfare centers in Chiba and Kanagawa prefectures, in the Kanto Region of Japan. None of the dogs exhibited any specific veterinary health abnormalities in their medical data. The age was not known for most animals, but samples were generally collected from adult dogs. In Chiba, the number of female and male dogs was 23 and 34, respectively; sex information was not available for 4 dogs. In Kanagawa, the number of female and male dogs was 25 and 38, respectively; sex information was not available for 14 dogs. In Chiba, the number of dogs belonging to different breeds was as follows: 45 hybrids, 6 Shiba Inu, 3 Beagle, 2 Toy Poodle, and 2 other breeds; breed information was not available for 3 dogs. In Kanagawa, it was: 17 hybrids, 8 Shiba Inu, 6 Toy Poodle, 5 Beagle, 4 Miniature Dachshund, and 23 other breeds; breed information was not available for 14 dogs. In Chiba, the dogs were introduced into animal welfare centers for the following reasons: 51 dogs were captured, including stray dogs; 5 dogs were abandoned; and information was not available for 5 dogs. In Kanagawa, the reasons were: 17 dogs were lost; 2 dogs were abandoned; and information was not available for 58 dogs.

The fecal samples were collected using a sterilized swab from naturally excreted feces. The portion in contact with the ground was not collected. Duplicate samples from the same animal were not collected. The fecal samples were preserved in Carry-Blair transport medium (Nissui Pharmaceutical Co., Ltd., Tokyo, Japan), stored at 4 °C, and transported to the laboratory for *E. coli* culture immediately.

### Detection of *E. coli*

The fecal samples were resuspended in sterilized saline solution and smeared onto an XM-G agar plate (Nissui Pharmaceutical Co., Ltd.) using a platinum loop. The plates were cultured under aerobic conditions at 35 °C for 20 h. After incubation, β-glucuronidase-positive colonies (a biochemical characteristic of *E. coli*) were selected and purified in nutrient agar (Eiken Chemical Co., Ltd., Tokyo, Japan). The selected colonies were identified as *E. coli* by polymerase chain reaction according to an established method^27^.

### Drug-susceptibility profile testing

The disk diffusion method, based on the performance standards issued by the CLSI^14^, was used to test the susceptibility of *E. coli* isolates toward all drugs except LVFX. Mueller–Hinton agar and antimicrobial susceptibility test discs (Sencsi-Disc) were purchased from BD Biosciences (Franklin Lakes, NJ, USA). The dry Eiken plate (Eiken Chemical Co., Ltd.), which uses the broth microdilution method based on the performance standards issued by the CLSI, was used for susceptibility testing of only LVFX (Table 1). Results of the antimicrobial susceptibility test were indicated as S, I, or R. *E. coli* ATCC25922 and *Pseudomonas aeruginosa* ATCC27853 (both from American Type Culture Collection, Manassas, VA, USA) were used as control strains.

### Chromosomal DNA and plasmid DNA extraction

PrepManUltra sample preparation reagent (Thermo Fisher Scientific, Waltham, MA, USA) was used for chromosomal DNA extraction. The Mini Plus Plasmid DNA Extraction System (Viogen-Bio Tek Corporation, Taipei, Taiwan) was used for plasmid DNA extraction.

### Detection of drug-resistance genes by PCR and DNA sequencing

Eighteen samples of multidrug-resistant *E. coli* were subjected to genetic testing to predict the mechanism of drug resistance. One of the strains (sample No. 16C1) presented poor growth; therefore, 17 samples were tested. *E. coli* that showed resistance or intermediate resistance to β-lactam antibiotics were analyzed for *blaTEM*, *blaSHV*, and *AmpC* (*bla CMY/MOX*, *bla CMY/LAT*, *bla DHA*, *bla ACC*, *bla ACT-1/MIR-1*, and *bla FOX*) genes^28,29^. In addition to this, we analyzed the *CTX-M* genes (*bla CTX-M-1-group*, *bla CTX-M-2-group*, *blaCTX-M-8-group*, and *bla CTX-M-9-group*) in *E. coli* that showed third-generation cephalosporin resistance or intermediate resistance^30^ and carbapenemase genes (*bla IMP-1*, *bla IMP-2*, *bla VIM-2*, *bla KPC-2*, *bla GES*, and *bla NDM-1*) in carbapenem-resistant *E. coli*^31–35^. Aminoglycoside antibiotic resistance and intermediate *E. coli* were analyzed for aminoglycoside resistance 16S rRNA methylases genes (*armA* and *rmtB*) and aminoglycoside-modifying enzyme genes (*Aac(6′)-Ib*, *Ant(3″)-Ia*, *Aph(3′)-Ia*, and *Aac(3)-II*)^36,37^. Quinolone-resistant and intermediate-resistant *E. coli* were analyzed for quinolone-resistance genes (*qnrA*, *qnrB*, *qnrC*, *qnrD*, *qnrS*, *qepA*, *oqxAB*, and *aac(6')-lb-cr*)^38^. The antibiotic resistance genes mentioned above were analyzed using the extracted plasmid DNA as a template to amplify the target region by PCR, followed by sequencing to decipher the nucleotide sequence and homology search by BLAST (https://blast.ncbi.nlm.nih.gov/Blast.cgi).). PCR and DNA sequencing analysis using chromosomal DNA as the template were performed to examine mutations in QRDR in quinolone-resistant and intermediate-resistant strains. In the DNA gyrase subunit A gene (*gyrA*), the mutations at 83S and 87D were analyzed^39^. In topoisomerase IV gene (*parC*), the mutations at 80S and 84 glutamic acid (E) were analyzed^40^. The primers used for the amplification of each gene and the references are shown in Table 3. The PCR conditions were based on the conditions described in the references, and the Multiplex PCR Kit (Takara Bio, Kyoto, Japan) was used for PCR. The ProFlex PCR System (Thermo Fisher Scientific) was used as the thermal cycler for PCR. The PCR amplification product was treated with Illustra ExoProStar (Cytiva, Marlborough, MA, USA) to remove unwanted nucleotides. The primers used for sequencing were the primers used for PCR amplification. DNA sequencing was outsourced to a specialized external organization (Fasmac Co., Ltd., Kanagawa, Japan). The nucleotide sequences were determined by the direct sequencing of PCR products, performed by Sanger sequencing on a 3730xl DNA Analyzer (Thermo Fisher Scientific) using the BigDye Terminator and BigDye XTerminator Purification Kit (Thermo Fisher Scientific)^41^.

**Table 3:** Primers used for amplification of drug-resistance genes.

Resistance mechanisms	Gene	Primer name	Sequence [5' → 3']	References no
β-lactamase	*bla TEM*	*TEM_F*	TCGTGTCGCCCTTATTCCCTTTTT	^28^
		*TEM_R*	GCGGTTAGCTCCTCCGGTCCTC	
	*bla SHV*	*SHV_F*	GTGGATGCCGGTGACGAACAGC	^28^
		*SHV_R*	TGGCGCAAAAAGGCAGTCAATCCT	
	*bla CTX-M-1-group*	*CTX-1_F*	CCCATGGTTAAAAAATCACTG	^30^
		*CTX-1_R*	CCGTTTCCGCTATTACAAAC	
	*bla CTX-M-2-group*	*CTX-2_F*	ATGATGACTCAGAGCATTCGC	^30^
		*CTX-2_R*	TCGCTCCATTTATTGCATCA	
	*blaCTX-M-8-group*	*CTX-8_F*	ATGTTAATGACGACAGCCTGTG	^30^
		*CTX-8_R*	CCGGTTTTATCCCCGACA	
	*bla CTX-M-9-group*	*CTX-9_F*	GATTGACCGTATTGGGAGTTT	^30^
		*CTX-9_R*	TATTGAGAGTTACAGCCCTTCG	
	*bla IMP-1*	*IMP1_F*	CTACCGCAGCAGAGTCTTTG	^31^
		*IMP1_R*	AACCAGTTTTGCCTTACAAT	
	*bla IMP-2*	*IMP2_F*	GTGTATGCTTCCTTTGTAGC	^32^
		*IMP2_R*	CAATCAGATAGGCGTCAGTGT	
	*bla VIM-2*	*VIM_F*	ATGGTGTTTGGTCGCATATC	^33^
		*VIM_R*	TGGGCCATTCAGCCAGATC	
	*bla KPC-2*	*KPC_F*	ATGTCACTGTATCGCCGTCT	^34^
		*KPC_R*	TTTTCAGAGCCTTACTGCCC	
	*bla GES*	*GES_F*	GTTTTGCAATGTGCTCAACG	^34^
		*GES_R*	TGCCATAGCAATAGGCGTAG	
	*bla NDM-1*	*NDM1_F*	CTGAGCACCGCATTAGCC	^35^
		*NDM1_R*	GGGCCGTATGAGTGATTGC	
	*bla CMY/MOX*	*MOXM_F*	GCTGCTCAAGGAGCACAGGAT	^29^
		*MOXM_R*	CACATTGACATAGGTGTGGTGC	
	*bla CMY/LAT*	*CITM_F*	TGGCCAGAACTGACAGGCAAA	^29^
		*CITM_R*	TTTCTCCTGAACGTGGCTGGC	
	*bla DHA*	*DHAM_F*	AACTTTCACAGGTGTGCTGGGT	^29^
		*DHAM_R*	CCGTACGCATACTGGCTTTGC	
	*bla ACC*	*ACCM_F*	AACAGCCTCAGCAGCCGGTTA	^29^
		*ACCM_R*	TTCGCCGCAATCATCCCTAGC	
	*bla ACT-1/MIR-1*	*EBCM_F*	TCGGTAAAGCCGATGTTGCGG	^29^
		*EBCM_R*	CTTCCACTGCGGCTGCCAGTT	
	*bla FOX*	*FOXM_F*	AACATGGGGTATCAGGGAGATG	^29^
		*FOXM_R*	CAAAGCGCGTAACCGGGATTGG	
Aminoglycoside resistance 16S rRNA methylases	*armA*	*armA_F*	GGTGCGAAAACAGTCGTAGT	^36^
		*armA_R*	TCCTCAAAATATCCTCTATGT	
	*rmtB*	*rmtB_F*	ATGAACATCAACGATGCCCT	^36^
		*rmtB_R*	CCTTCTGATTGGCTTATCCA	
Aminoglycoside modifying enzyme	*Aac(6′)-Ib*	*Aac(6′)-I-F*	AAACCCCGCTTTCTCGTAGC	^37^
		*Aac(6′)-I-R*	AAACCCCGCTTTCTCGTAGC	
	*Ant(3″)-Ia*	*Ant(3″)-F*	CCGGTTCCTGAACAGGATC	^37^
		*Ant(3″)-R*	CCCAGTCGGCAGCGACATC	
	*Aph(3′)-Ia*	*Aph(3′)-F*	CAAGATGGATTGCACGCAGG	^37^
		*Aph(3′)-R*	TTCAGTGACAACGTCGAGCA	
	*Aac(3)-II*	*Aac(3)-II-F*	GCTCGGTTGGATGACAAAGC	^37^
		*Aac(3)-II-R*	AGGCGACTTCACCGTTTCTT	
Quinolone resistance protein	*qnrA*	*qnrA_F*	AGAGGATTTCTCACGCCAGG	^38^
		*qnrA_R*	GCAGCACTATKACTCCCAAGG	
	*qnrB*	*qnrB_F*	GGMATHGAAATTCGCCACTG	^38^
		*qnrB_R*	TTTGCYGYYCGCCAGTCGAA	
	*qnrC*	*qnrC_F*	GGGTTGTACATTTATTGAATC	^38^
		*qnrC_R*	TCCACTTTACGAGGTTCT	
	*qnrD*	*qnrD_F*	CGAGATCAATTTACGGGGAATA	^38^
		*qnrD_R*	AACAAGCTGAAGCGCCTG	
	*qnrS*	*qnrS_F*	GCAAGTTCATTGAACAGGCT	^38^
		*qnrS_R*	TCTAAACCGTCGAGTTCGGCG	
	*qepA*	*qepA_F*	CTGCAGGTACTGCGTCATG	^38^
		*qepA_R*	CGTGTTGCTGGAGTTCTTC	
	*oqxA*	*oqxA_F*	GACAGCGTCGCACAGAATG	^38^
		*oqxA_R*	GGAGACGAGGTTGGTATGGA	
	*oqxB*	*oqxB_F*	CGAAGAAAGACCTCCCTACCC	^38^
		*oqxB_R*	CGCCGCCAATGAGATACA	
	*aac(6′)-Ib*	*aac_F*	TTGCGATGCTCTATGAGTGGCTA	^38^
		*aac_R*	CTCGAATGCCTGGCGTGTTT	
Mutation of the quinolone resistance-determining regions	*gyrA*	*STGYRA_F*	TGTCCGAGATGGCCTGAAGC	^39^
		*STGYRA_R*	CGTTGATGACTTCCGTCAG	
	*parC*	*parC_F*	TGTATGCGATGTCTGAACTG	^40^
		*parC_R*	CTCAATAGCAGCTCGGAATA	

### Statistical analysis

The sex differences in the rate of S, I, and R were evaluated using the chi-square test. SPSS (version 19, IBM Japan, Tokyo, Japan) was used for the analysis. The statistical significance level was set to 5%.

The 95% CI of resistance rates were calculated using the Agresti-Coull method.

## Data Availability

The datasets generated and analyzed during the current study are available from the corresponding author on reasonable request.

## References

[1] Chen JW (2020). Antibiotic-resistant *Escherichia coli* and sequence Type 131 in fecal colonization in dogs in Taiwan. Microorganisms.

[2] Gwenzi, W. *et al.* Insects, rodents, and pets as reservoirs, vectors, and sentinels of antimicrobial resistance. *Antibiotics (Basel)* **10**, 68 (2021). 10.3390/antibiotics10010068 PMC7826649 33445633

[3] Pomba C (2017). Public health risk of antimicrobial resistance transfer from companion animals. J. Antimicrob. Chemother..

[4] Joosten, P. *et al.* Antimicrobial usage and resistance in companion animals: A cross-sectional study in three European countries. *Antibiotics (Basel)* **9**, 87 (2020). 10.3390/antibiotics9020087 PMC7175148 32079072

[5] Kidsley AK (2020). Companion animals are spillover hosts of the multidrug-resistant human extraintestinal *Escherichia coli* pandemic clones ST131 and ST1193. Front. Microbiol..

[6] Toombs-Ruane LJ (2020). Carriage of extended-spectrum-beta-lactamase- and AmpC beta-lactamase-producing *Escherichia coli* strains from humans and pets in the same households. Appl. Environ. Microbiol..

[7] Wedley AL (2017). Carriage of antimicrobial resistant *Escherichia coli* in dogs: Prevalence, associated risk factors and molecular characteristics. Vet. Microbiol..

[8] Sevilla E (2020). Antimicrobial resistance among canine enteric *Escherichia coli* isolates and prevalence of attaching-effacing and extraintestinal pathogenic virulence factors in Spain. Acta Vet. Hung..

[9] Bortolami A (2019). Diversity, virulence, and clinical significance of extended-spectrum beta-lactamase- and pAmpC-producing *Escherichia coli* from companion animals. Front. Microbiol..

[10] World Health Organization. Global action plan on antimicrobial resistance. Available at: https://www.who.int/iris/bitstream/10665/193736/1/9789241509763_eng.pdf?ua=1 (2015). 10.7196/samj.9644 26242647

[11] Government of Japan. National action plan on antimicrobial resistance (AMR) 2016–2020. Available at: https://www.mhlw.go.jp/file/06-Seisakujouhou-10900000-Kenkoukyoku/0000138942.pdf (2016). 10.35772/ghm.2019.01017 PMC7731179 33330758

[12] Interagency Coordination Group on antimicrobial resistance (IACG). No time to wait: Securing the future from drug-resistant infections in. *Report to the Secretary-General of the United Nations*. Available at: https://www.who.int/antimicrobial-resistance/interagency-coordination-group/final-report/en. (2019).

[13] Ministry of the Environment & Government of Japan. Guideline for adoption support. Available at: https://www.env.go.jp/nature/dobutsu/aigo/2_data/pamph/support/02.pdf (2006).

[14] Clinical and Laboratory Standards Institute (CLSI). Performance standards for antimicrobial susceptibility testing; twenty-fourth informational supplement (M100-S24). Available at: Available at:https://www.researchgate.net/file.PostFileLoader.html?id=59202a0696b7e4d462166956&assetKey=AS%3A496054988533760%401495280134033 (2014).

[15] National Veterinary Assay Laboratory & Ministry of Agriculture, Forestry and Fisheries of Japan (MAFF). FY 2018 antimicrobial resistance monitoring survey of bacteria derived from healthy companion animals (dogs and cat). Available at: https://www.maff.go.jp/nval/yakuzai/pdf/H30kenkocyousa20200212.pdf (2020).

[16] Schmidt VM (2018). Routine antibiotic therapy in dogs increases the detection of antimicrobial-resistant faecal *Escherichia coli*. J. Antimicrob. Chemother..

[17] Poirel, L. *et al*. Antimicrobial resistance in *Escherichia coli*. *Microbiol. Spectr.* **6** (2018). 10.1128/microbiolspec.ARBA-0026-2017. 10.1128/microbiolspec.arba-0026-2017 PMC11633601 30003866

[18] Kawamura K (2017). ESBL-producing *Escherichia coli* and its rapid rise among healthy people. Food Saf. (Tokyo).

[19] Liao XP (2015). Characterization of CTX-M-14-producing *Escherichia coli from* food-producing animals. Front. Microbiol..

[20] Cormier A (2019). Diversity of CTX-M-positive *Escherichia coli* recovered from animals in Canada. Vet. Microbiol..

[21] Tamang MD (2012). Molecular characterization of extended-spectrum-β-lactamase-producing and plasmid-mediated AmpC β-lactamase-producing *Escherichia coli* isolated from stray dogs in South Korea. Antimicrob. Agents Chemother..

[22] Umeda K, Hase A, Matsuo M, Horimoto T, Ogasawara J (2019). Prevalence and genetic characterization of cephalosporin-resistant Enterobacteriaceae among dogs and cats in an animal shelter. J. Med. Microbiol..

[23] Tamang MD (2012). Prevalence of plasmid-mediated quinolone resistance determinants among *Escherichia coli* isolated from food animals in Korea. Foodborne Pathog. Dis..

[24] Chung YS (2017). Mechanisms of quinolone resistance in *Escherichia coli* isolated from companion animals, pet-owners, and non-pet-owners. J. Vet. Sci..

[25] Guillard T (2016). Characterization of quinolone resistance mechanisms in Enterobacteriaceae recovered from diseased companion animals in Europe. Vet. Microbiol..

[26] Umeda, K. *et al*. Prevalence and mechanisms of fluoroquinolone-resistant Escherichia coli among sheltered companion animals. *Access Microbiol.* **2,** acmi000077 (2020). 10.1099/acmi.0.000077 PMC7525055 33062936

[27] Wang RF, Cao WW, Cerniglia CE (1996). PCR detection and quantitation of predominant anaerobic bacteria in human and animal fecal samples. Appl. Environ. Microbiol..

[28] Arlet G, Philippon A (1991). Construction by polymerase chain reaction and intragenic DNA probes for three main types of transferable β-lactamases (TEM, SHV, CARB) [corrected]. FEMS Microbiol. Lett..

[29] Pérez-Pérez FJ, Hanson ND (2002). Detection of plasmid-mediated AmpC beta-lactamase genes in clinical isolates by using multiplex PCR. J. Clin. Microbiol..

[30] Jeong SH (2005). Dissemination of transferable CTX-M-type extended-spectrum β-lactamase-producing *Escherichia coli* in Korea. J. Appl. Microbiol..

[31] Senda K (1996). PCR detection of metallo-beta-lactamase gene (blaIMP) in gram-negative rods resistant to broad-spectrum beta-lactams. J. Clin. Microbiol..

[32] Riccio ML (2000). Characterization of the metallo-β-lactamase determinant of *Acinetobacter baumannii* AC-54/97 reveals the existence of bla_IMP_ allelic variants carried by gene cassettes of different phylogeny. Antimicrob. Agents Chemother..

[33] Poirel L (2000). Characterization of VIM-2, a carbapenem-hydrolyzing metallo-beta-lactamase and its plasmid- and integron-borne gene from a Pseudomonas aeruginosa clinical isolate in France. Antimicrob. Agents Chemother..

[34] Bradford PA (2004). Emergence of carbapenem-resistant Klebsiella species possessing the class A carbapenem-hydrolyzing KPC-2 and inhibitor-resistant TEM-30 beta-lactamases in New York City. Clin. Infect. Dis..

[35] Pfeifer Y (2011). Molecular characterization of blaNDM-1 in an *Acinetobacter baumannii* strain isolated in Germany in 2007. J. Antimicrob. Chemother..

[36] González-Zorn B (2005). armA and aminoglycoside resistance in *Escherichia coli*. Emerg. Infect. Dis..

[37] Shi, Y. *et al*. A rapid and accurate method for the detection of four aminoglycoside modifying enzyme drug resistance gene in clinical strains of *Escherichia coli* by a multiplex polymerase chain reaction. *PeerJ* **8**, e8944 (2020). 10.7717/peerj.8944 PMC7153551 32309051

[38] Chen X (2012). Prevalence of qnr, aac(6′)-Ib-cr, qepA, and oqxAB in *Escherichia coli* isolates from humans, animals, and the environment. Antimicrob. Agents Chemother..

[39] Giraud, E., Brisabois, A., Martel, J. L. & Chaslus-Dancla, E. Comparative studies of mutations in animal isolates and experimental in vitro- and in vivo-selected mutants of *Salmonella* spp. suggest a counterselection of highly fluoroquinolone-resistant strains in the field. *Antimicrob. Agents Chemother.* **43**, 2131–2137 (1999). 10.1128/aac.43.9.2131 PMC89435 10471553

[40] Everett MJ, Jin YF, Ricci V, Piddock LJ (1996). Contributions of individual mechanisms to fluoroquinolone resistance in 36 Escherichia coli strains isolated from humans and animals. Antimicrob. Agents Chemother..

[41] Sanger, F., Nicklen, S. & Coulson, A. R.. DNA sequencing with chain-terminating inhibitors. *Proc. Natl Acad. Sci. U. S. A.* **74**, 5463–5467 (1977). 10.1073/pnas.74.12.5463 PMC431765 271968

